# Size-Selective Capturing of Exosomes Using DNA Tripods

**DOI:** 10.1021/jacs.3c11067

**Published:** 2024-04-03

**Authors:** Ryosuke Iinuma, Xiaoxia Chen, Takeya Masubuchi, Takuya Ueda, Hisashi Tadakuma

**Affiliations:** † Graduate School of Frontier Science, The University of Tokyo, Chiba 277-8562, Japan; ‡ JSR Corporation, Ibaraki, 305-0841, Japan; § School of Life Science and Technology, ShanghaiTech University, Shanghai 201210, People’s Republic of China; ∥ Department of Cell and Developmental Biology, School of Biological Sciences, University of California San Diego, La Jolla, California 92093, United States; ⊥ Graduate School of Science and Engineering, Waseda University, Tokyo 162-8480, Japan; # Gene Editing Center, School of Life Science and Technology, ShanghaiTech University, Shanghai 201210, People’s Republic of China

## Abstract

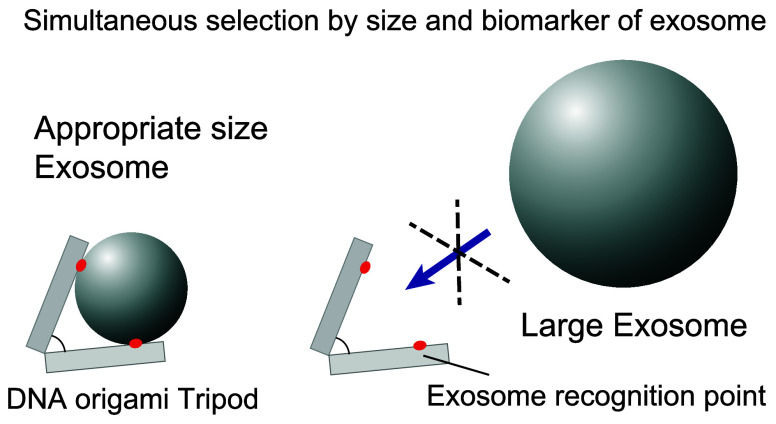

Fractionating and characterizing target samples are fundamental to the analysis of biomolecules. Extracellular vesicles (EVs), containing information regarding the cellular birthplace, are promising targets for biology and medicine. However, the requirement for multiple-step purification in conventional methods hinders analysis of small samples. Here, we apply a DNA origami tripod with a defined aperture of binders (e.g., antibodies against EV biomarkers), which allows us to capture the target molecule. Using exosomes as a model, we show that our tripod nanodevice can capture a specific size range of EVs with cognate biomarkers from a broad distribution of crude EV mixtures. We further demonstrate that the size of captured EVs can be controlled by changing the aperture of the tripods. This simultaneous selection with the size and biomarker approach should simplify the EV purification process and contribute to the precise analysis of target biomolecules from small samples.

## Introduction

Extracellular vesicles (EVs) are nanosized lipid-bilayer-enclosed membrane vesicles released from almost all mammalian cells.^1,2^ EVs contribute to intercellular communication by carrying biomolecular cargos such as miRNAs, mRNAs, and proteins.^3−5^ These functions are being vigorously investigated for potential clinical applications.^6−8^

Isolating a subpopulation of EVs is potentially significant for clinical applications since individual EVs contain information regarding the cell status of their birthplace. Conventional EV isolation methods utilize differential ultracentrifugation, although recently alternative methods have been proposed, including precipitation, size exclusion chromatography, ion chromatography, and immuno-affinity.^9^ Moreover, multiple purification methods can be combined to improve isolation resolution. Recent EV analysis unveiled the detailed characteristics of EVs, demonstrating that EV subpopulations reflect the birthplace. Such analysis suggests that EVs might be a potential biomaker for accurate classification of diseases such as cancer.^6,7^ For such purposes, the size and surface markers of EVs are considered as key features for their isolation and identification.^9^ However, limited methods exist for size-dependent isolation of EVs, and methods simultaneously harnessing the size separation and identification of surface protein markers have been rarely demonstrated. Furthermore, the combination of multiple purification methods of the past approaches has made it difficult to precisely discriminate EVs, causing potential misclassification of similarly sized EVs with diverse cargos and surface modifications.

DNA nanotechnology offers nanometer-sized, well-ordered precise structures.^10−16^ This technology has been used for precise alignment of functional binders, such as aptamers, antibodies, chemical compounds, and nanoparticles for specific capture of target molecules.^17−23^ Particularly, 3D DNA origami has extensionally provided user-defined features and enabled specific virus capture or cell recognition.^24−29^ However, the application of this technology is limited for EV capture, especially as past approaches require a large number of nanostructures to capture an EV, limiting size discrimination capability.^30−32^ Here, we demonstrate a novel method for selective capture of EVs that have a user-defined vesicle size and surface protein marker. Using a geometrical structural feature of 3D DNA origami, we captured EVs of a specific size from samples containing a broad distribution of vesicles. Our method should be the basis for future smart devices for selective capture in research and clinical applications.

## Results and Discussion

**Figure 1:**
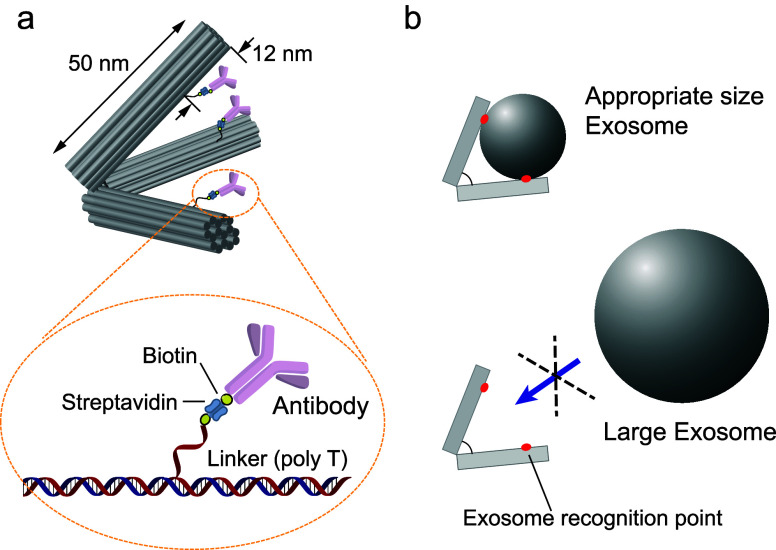
Schematic illustration of EV capture using DNA tripods. (a) Design of DNA origami tripod for EV capture. Antitetraspanin antibody was introduced at polyT strand ends on each arm of the tripod via biotin/streptavidin conjugation. (b) Controlling the angle of tripod structure permits access to EVs of specific sizes with recognition points set inside the structure. Detailed specification of size selectivity is described in Figures S2 and S3.

We used a tripod which has a three-arm-junction structure with a defined hinge angle as the base structure (Figures 1and S1–S3). We reasoned that a specific angle structure would limit the accessibility of the binder and permit the size-selective capture of target vesicles in two different ways. First, the specific angle of the tripod defined the aperture of the tripod arms, and only particles smaller than a certain diameter can access the binders buried inside arm, whereas particles larger than the aperture cannot. Second, kinetic selection (a fast overall *k*_off_ from the tripod) reduces the maintenance probability of smaller EVs that can bind to only one or two of the three binders. Therefore, only EVs that fit within the aperture will be retained, facilitating size-selective capture. We attached a biotinylated antibody to each arm using an avidin–biotin interaction, resulting in a tripod with three antibodies. We designed antibody attachment sites to be buried inside the tripod (12 nm from the tip of tripod arms, compared to IgG size of ∼ 15 nm), thus limiting their interactions with EVs smaller than the tripod aperture.

**Figure 2:**
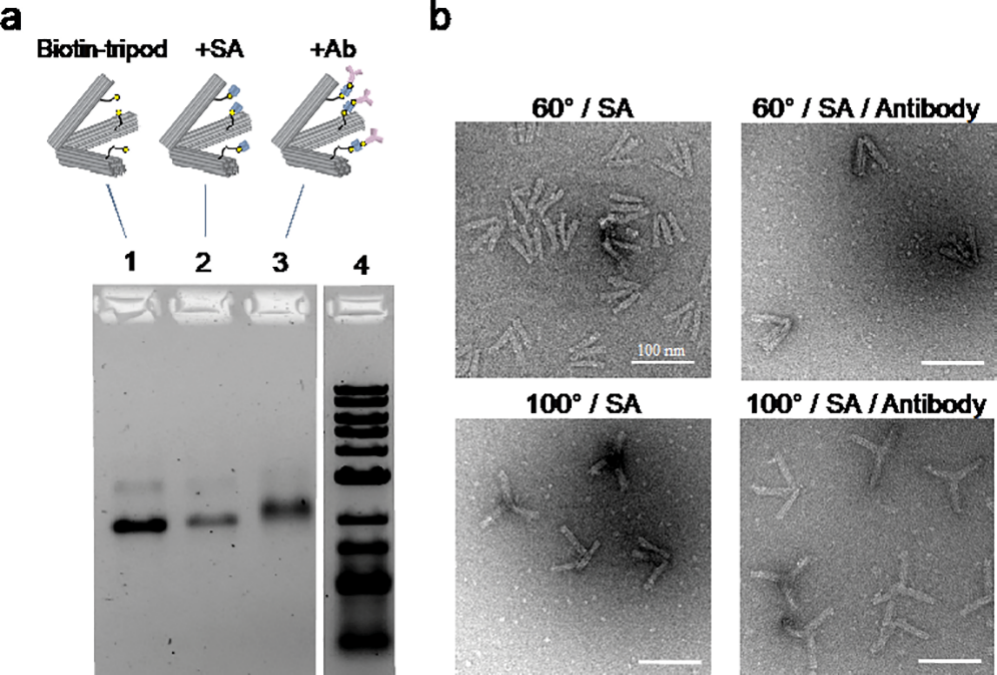
Self-assembly of DNA origami tripods using antitetraspanin antibody. (a) Agarose gel electrophoresis of a DNA origami tripod. Lane 1 is a DNA origami tripod with 60°–60°–60° angles and introduced with three biotin sites. Lane 2 is a DNA origami tripod structure introduced with streptavidin (SA). Lane 3 is DNA origami tripod structure introduced with anti-CD9 antibody. (b) TEM images of DNA tripod. (upper left) 60°–60°–60° DNA tripod with biotin/streptavidin sites (lane 2 in the gel). (upper right) 60°–60°–60° DNA tripod with biotin/SA/anti-CD-9 antibody (lane 3 in the gel. mean ± SD is 50 ± 9.4 deg, *n* = 55, see Figure S4). (lower left) 100°–100°–100° DNA tripod with biotin/streptavidin sites. (lower right) 100°–100°–100° DNA tripod with biotin/SA/anti-CD-9 antibody.

We first confirmed the attachment of antibodies by a gel shift assay (Figure 2). As expected, the band of tripod shifted upward with the attachment of streptavidin. Antibody attachment further shifted the band, showing successful antibody attachment. We further confirmed antibody attachment by negative staining and visualization using an electron microscope. The negative stain images clearly showed the attachment of antibody on a tripod (designed with defined angle: 60°–60°–60° and 100°–100°–100°) (Figures 2b and S4). The labeling ratio of antibody was confirmed by fluorescent intensity using the gel shift assay (Figure S5). To estimate the number of antibodies attached on the tripod, we used fluorescently labeled antibody and fluorescently labeled streptavidin, and measured the band intensity of antibody-conjugated tripods (see Supplemental Methods for detail). We found that approximately 3 antibodies were present on each DNA tripod, suggesting that all of the antibody attachment sites were occupied.

**Figure 3:**
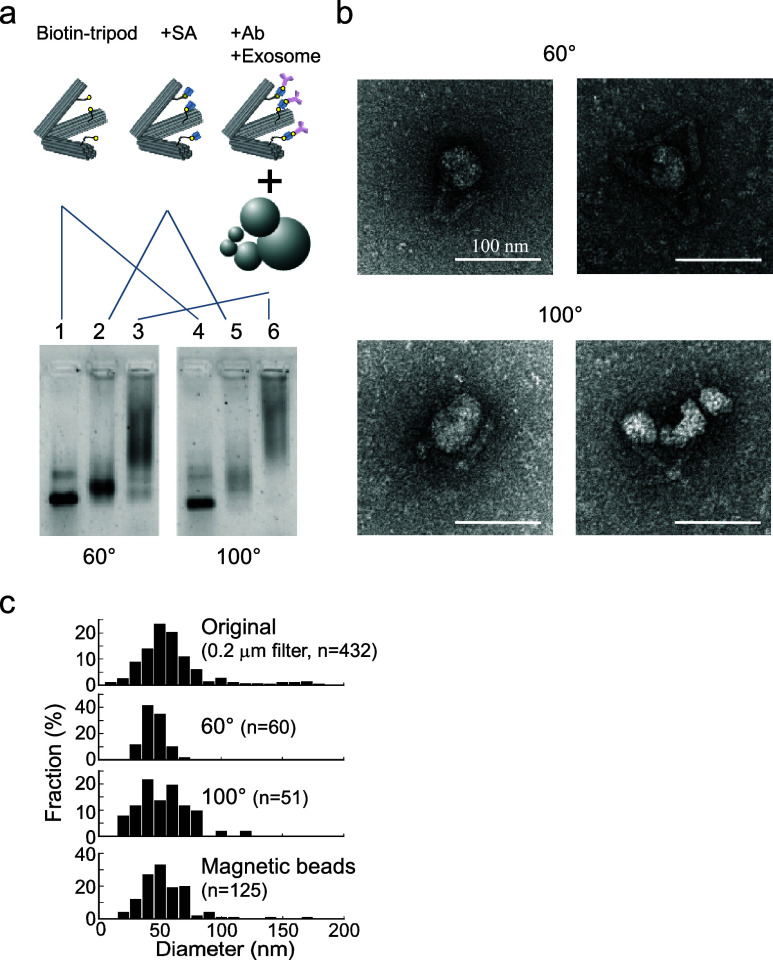
EV capture with a DNA tripod and distribution analysis. (a) Gel electrophoresis analysis of DNA tripods and EVs. Lanes 1–3 show the 60°–60°–60° DNA tripod, lanes 4–6 show the 100°–100°–100° DNA tripod. Lane 3 and 6 report the reaction of EVs and DNA tripods, with 60°–60°–60° and 100°–100°–100° angles, respectively. (b) (upper) TEM images of reaction mixture of EVs and 60°–60°–60° DNA tripod. (lower) TEM images of reaction mixture of EVs and the 100°–100°–100° DNA tripod. (c) Size distribution of exosomes: (upper) original EVs prepared from HT-29 cell supernatant (mean ± SD: 65 ± 36 nm); (second) EVs of captured with the 60°–60°–60° DNA tripod (47 ± 8.7 nm); (third) EVs captured with the 100°–100°–100° DNA tripod (55 ± 21 nm) and (bottom) EVs of captured with an anti CD-9 antibody conjugated magnetic beads (60 ± 21 nm). *p*-Values of Kolmogorov–Smirnov test that compare the observed and original distribution (0.2 μm filter sample) were <0.01, > 0.1, and >0.1 for the 60°–60°–60° DNA tripod, 100°–100°–100° DNA tripod, and magnetic bead sample, respectively. Furthermore, the Kruskal–Wallis test showed that there were significant changes in all groups (*p* = 4.6 × 10^–6^). The *p*-values of Dunn’s test that compare the observed and original distribution (0.2 μm filter sample) were 3 × 10^–6^, 0.59, and 1.0 for the 60°–60°–60° DNA tripod, 100°–100°–100° DNA tripod, and magnetic bead sample, respectively.

After confirming the assembly of the antibody-attached tripod (hereafter Ab-Tripod), we mixed it with model EVs. To generate a model crude EV sample, we prepared EVs from the HT-29 cell supernatant and filtered it to remove cell debris. After incubation with EVs, the band of the Ab-Tripod considerably shifted to a larger molecular size and showed smearing, suggesting complex of EVs and Ab-Tripods were formed. We also optimized the antibody to use anti-CD9 antibody, where the optimal antibody might be different for different origins (birthplaces) of exosomes (Figures S6 and S7). Interestingly, the position of the smear obtained by gel analysis differed depending on the hinge angle of the Ab-Tripod. For example, the 100°–100°–100° Ab-Tripod yielded a smear at a relatively larger molecular size than the 60°–60°–60° Ab-Tripod (Figure 3a), suggesting the formation of larger complexes.

To investigate the morphology of these complexes, we further subjected reaction mixtures to TEM analysis. As expected, both types of Ab-Tripods (with different apertures) showed clear evidence of EV capture inside the tripod arms. We found that Ab-Tripods only captured EVs with sizes smaller than the designed aperture, especially for Ab-tripods with 60°–60°–60°. Moreover, we identified several distinct binding modes, including single and multiple EVs associated with a single Ab-Tripod or a single EV associated with multiple Ab-Tripods (Figures 3b and S8). Particularly, the 100°–100°–100° Ab-Tripod tended to capture multiple numbers of EVs within its arms having a widely opened aperture design (Figure 3b **right**). This variety in complex type might result in the observed smearing of agarose gel.

To further verify the concept of size-selective capture of EVs with Ab-Tripods, we measured the size of captured EVs using TEM images. The sizes of EVs with the 60°–60°–60° Ab-Tripod showed a sharper distribution with 45 nm as the maximum frequency (Figure 3c), which satisfies the designed aperture size of Ab-Tripods (*p* < 0.01, Kolmogorov–Smirnov (KS) test). The captured size (47 ± 8.7 nm) is slightly smaller than the selective size based on design (54–71 nm, Figures S2 and S3). Moreover, using model particles (SA-coated magnetic beads of 50 and 150 nm), we confirmed that the 60°–60°–60° Ab-Tripod can capture only 50 nm but not 150 nm model particles (Figure S9). On one hand, Monte Carlo simulations support our view that target-sized EVs can remain longer than smaller particles in multibinder systems (Figure S10). Meanwhile, the size distribution of EVs captured by the 100°–100°–100° Ab-Tripod was considerably wide (Figure 3c). The size distribution of captured EVs was almost the same as original EVs, although the frequency of the larger EVs was slightly reduced (Figure 3c, *p* > 0.1, KS test). These results demonstrated that the 100°–100°–100° Ab-Tripod is unable to sort specific size of EVs, suggesting that the balance between the aperture and target size is important for the tripod design. Future affinity tuning of the binder (Ab in this study) would achieve a better kinetic selection and should improve the rational design capability of the capture system.^22,33^ We also recognized that some of the Ab-Tripods were broken, which might be caused by contaminants (e.g., DNase) in the exosome solution. Further design improvement,^34^ introduction of internal cross-linking (e.g., thymine dimer introduced by UV irradiation,^35−37^ and chemical modification of DNA origami (e.g., PEG coating^38^) will improve the toughness of DNA tripods.

Next, we investigated the EV size distribution captured by antibody-conjugated magnetic beads, which is one of the gold standards for small-scale EV purification. Commercially available anti-CD9 antibody-conjugated magnetic bead kit was used, with procedures performed according to the manufacturer’s instruction and using the provided reagents (e.g., release buffer). The observed distribution of the released EVs was similar to the distribution of original EVs (Figure 3c, *p* > 0.1, KS test), suggesting that the magnetic bead method is not optimal for size-selective capturing of EVs.

We next investigated interaction kinetics between Ab-Tripods and EVs (Figures S11 and S12). Kinetic parameters were estimated from the fraction of the Ab-Tripod/EV complex to total Ab-Tripod for different periods up to 19 h after initial mixing. For the 60°–60°–60° Ab-Tripod, the fraction of Ab-Tripod with EVs reached 52% after 1 h, whereafter it gradually increased and showed a plateau at around 75% after 19 h. The same experiments were performed for a negative control condition, in which antimouse IgG 2a Ab-Tripod was used. In this negative control condition, the complex fraction reached around 10% after 1 h, and remained around 20% even after 19 h. For the 100°–100°–100° Ab-Tripod, the reaction showed a much faster rate. After mixing Ab-Tripod and EVs, a band for unreacted Ab-Tripods disappeared after 1 h and a large smear band appeared instead. These results showed that the binding speed of EVs to Ab-Tripod is highly sensitive to EV accessibility to the antibody present on the tripod.

**Figure 4:**
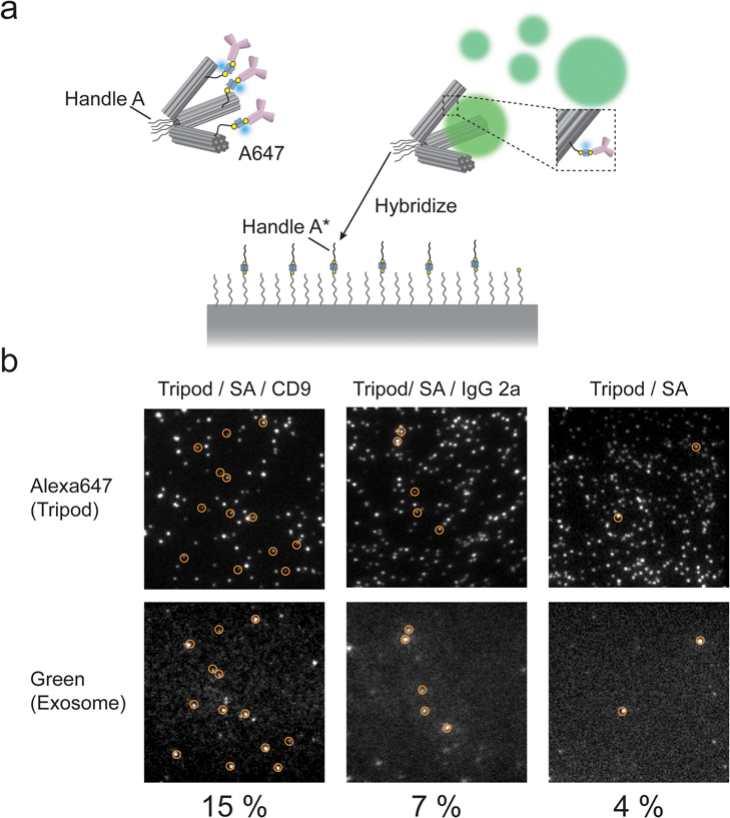
Capture of a single exosome by DNA tripods anchored onto a solid surface. (a) Schematic illustration of exosome capture using a single-molecule imaging microscope. (b) Co-localization of exosomes and 60°–60°–60° DNA tripods are shown in orange circles. Co-localization ratio of anti-CD-9 Ab-Tripod was around 15% (62 molecules/402 region of interests (ROIs)). By contrast, the colocalization ratio of control condition was low: 7% (29 molecules/419 ROIs) and 4% (18 molecules/435 ROIs) for antimouse IgG2a antibody and nonantibody introduced tripod, respectively.

Given the size-selective capture using Ab-Tripods, we next applied the principle to a solid-supported capture system for EVs. To immobilize Ab-Tripods on a solid surface, multiple short DNA strands were introduced to the vertex position of Ab-Tripods (Figure 4a). DNA strands complementary against Ab-Tripod vertex were anchored onto a PEG-coated glass surface via the biotin/streptavidin reaction.^39−41^ EVs were stained with green fluorescent dye using a commercially available kit, while tripods were labeled with red fluorescent dye. After mixing EVs and Ab-Tripods for 12 h, the reaction mixture was introduced into a chamber (Figure 4b). After the unreacted EVs were washed out, we measured the number of EVs bound to the glass surface using total internal reflection fluorescence (TIRF) microscopy. This was performed for three Ab-Tripod designs: anti-CD-9 Ab-Tripod, anti-IgG2a Ab-Tripod, and tripod without antibody.

Using single-molecule fluorescence microscopy, we distinguished between EVs specifically bound to Ab-Tripods and nonspecifically bound to the glass surface. We used colocalization as an indicator of specific binding; i.e., we compared the red fluorescent image (Alexa 647) of Ab-Tripods with that of green fluorescent image of EVs and defined colocalized fluorescent spots as an EV–Ab-Tripod complex.

We found that the colocalization ratio of anti-CD-9 Ab-Tripod was around 15% (62 molecules/402 region of interests (ROIs)). By contrast, the colocalization ratio of control condition was low: 7% (29 molecules/419 ROIs) and 4% (18 molecules/435 ROIs) for antimouse IgG2a antibody and tripod with no antibody, respectively. These results suggest that EVs can be captured and analyzed at the single-particle level using a solid support-based system (Figure 4b).

## Conclusion

Here, we constructed DNA origami nanodevices that have a defined aperture to permit the size- and biomarker-specific capture of EVs. Moreover, by integrating a variety of binders (e.g., antibodies/aptamers against proteins and lectin against sugar chains), this tripod-based system should be a versatile method to capture specific EVs with defined size and specific biomarker expression patterns. Toward these goals, we further need to explore the effect of key factors (kinetics of binders, combination of different binders (e.g., anti-CD9 and anti CD63), numbers, and layout of binders). This concept can be also applied to detect other types of biological molecules, such as protein complexes and protein–nucleic acid complexes, by changing aperture geometries and binder layouts. Conventional bead systems are based on the random-adsorption of binders, and therefore it is difficult to control the two- and three-dimensional layout of the binders, experiencing difficulties when used to capture specific targets in a precise manner. To compensate for the low specificity, tandem purification methods are conventionally used at the expense of sample loss. This trade-off between specificity and yield can be solved using the one-step purification capability of DNA origami-based system. Specifically, combining affinity design methods based on multibinder system,^22,25,33^ DNA origami approach should open the way to purify specific targets from small samples with high specificity, which is difficult to achieve with other systems. Moreover, future implementation of the mechanical actuator capability of DNA origami^42^ and integrated enzymatic systems on DNA origami^21^ will open the way to develop nanorobots that can analyze and/or treat target particles (e.g., EVs and cells) at the single-particle level.

## Supporting Information Available

Design of DNA tripod, quantification of antibodies integrated onto DNA tripods, kinetic analysis of interactions between exosomes and DNA tripods. Protocols for DNA tripod preparation protocol; conjugation of antibodies; exosome extraction; EV capture using DNA tripods; TEM observation; single-particle imaging (PDF)
