# Supplementary material for: Size-Selective Capturing of Exosomes Using DNA Tripods
Source: J Am Chem Soc. 2024 Apr 3;146(15):10293–8. doi: 10.1021/jacs.3c11067 (PMC11027911; doi:10.1021/jacs.3c11067)
Supplement: Supplementary file 1 — ja3c11067_si_001.pdf [file ja3c11067_si_001.pdf]

**Supplementary Information for**

# Size-selective capturing of exosomes using DNA tripods

Ryosuke Iinuma, Xiaoxia Chen, Takeya Masubuchi, Takuya Ueda and Hisashi Tadakuma

**This file includes:**

Supplementary Material and Methods

Supplementary Figures (Supplementary Fig. 1 to 12)

Supplementary Tables (Supplementary Table 1)

# Supplementary Material and Methods

## Contents

1. **DNA tripod preparation**
2. **Conjugation of A647 with anti-CD9 antibody**
3. **Quantification of antibodies integrated onto DNA tripods**
4. **Agarose gel electrophoresis**
5. **Exosome extraction**
6. **EV capture using DNA Tripods**
7. **TEM observation**
8. **Single-particle imaging**
9. **Quantification of antibodies introduced onto DNA tripods (related to Supplementary Fig. 5)**
10. **Model particle capture (related to Supplementary Fig. 9)**
11. **Simulation analysis of the interaction between exosomes and DNA tripods (related to Supplementary Fig. 10)**

## 1. DNA tripod preparation

DNA tripods were designed based on previous report[14] with caDNAno (ver 0.2.3). DNA strands required for the structure were synthesized by Integrated DNA Technologies (IDT). To assemble DNA tripod structures, DNA oligonucleotides were mixed with p8064 (Tilibit nanosystems) scaffold in a molar stoichiometric ratio of 30:1 for biotinylated nucleotides, and 5:1 for other strands in TE buffer (5 mM Tris, pH 8.0, and 1 mM EDTA) supplemented with 9 mM $MgCl_2$ (DNA Origami folding buffer). The final concentration

of p8064 scaffold was adjusted to 20 nM. The strand mixture was annealed in a PCR tube using fast linear cooling step from 80℃ to 65℃ over 1 hour, followed by a 24 hours linear cooling ramp from 64℃ to 24℃.

The annealed solution was purified with micro spin columns (Cytiva illustra S-300HR). The buffer of micro spin column was replaced with DNA Origami folding buffer before use. 40 μL of reaction solution (DNA Origami 5–6 nM) was applied to a column and centrifuged to facilitate purification. The flow through from the column was reapplied to a new column to remove excess DNA short strands. The obtained solution was analyzed with NanoDrop 3000 (Thermo) to record absorbance at 260 nm to determine the DNA tripod concentration.

For introducing anti-CD9, anti-CD63, and EpCAM antibody, Alexa Fluor 647 (A647) labeled streptavidin (SA) was added to the solution obtained from the former process in a molar stoichiometric ratio (3:1) against the binding sites of DNA tripod, and incubated for 30 min at room temperature. A 40 μL of the reaction solution was applied to a column to remove excess streptavidin. Biotinylated anti-CD9 (MBL, MEX001-6), anti-CD63 (MBL, MEX002-6), and anti-EpCAM (MBL, MEX004-6) were then added to the obtained flow-through in a molar stoichiometric ratio 1:1 to the binding sites of the DNA tripod, and the resulting mixture was incubated for 1 hour. This reaction solution was then used for subsequent exosomes capture processes without further purification. We note that when reducing the biotin sites from 3 to 1 per DNA Tripod, we also reduced the amount of SA and antibody to a molar stoichiometric ratio of 1:1 to the binding sites of the DNA tripod.

## 2. Conjugation of A647 with anti-CD9 antibody

For conjugating A647 to anti-CD9 antibody, an azide moiety was first attached to the heavy chain of the antibody and subsequently reacted with an sDIBO alkyne-labeled A647 through a copper-free click reaction. All reactions were performed using protocols provided by SiteClick antibody Azide modification kit and Click-iT sDIBO Alkyne for SiteClick antibody labeling kit (Invitrogen, Cat#S20026 and C20029, respectively).

## 3. Quantification of antibodies integrated onto DNA tripods

The number of antibodies on DNA tripods was estimated using gel analysis (see Supplemental Methods for details). Briefly, the fluorescent intensity of A647 labeled anti-CD9 antibody (A647-anti-CD9) was measured and the amount of A647-anti-CD9 was estimated using A647-labeled streptavidin as a reference.

## 4. Agarose gel electrophoresis

The reaction mixture was subjected to 1.0% agarose gel electrophoresis, in which the gel was supplemented with 10 mM $MgCl_2$ and pre-stained with SYBRSafe (Thermo). The gel was run in 0.5% TBE buffer with 10 mM $MgCl_2$ for 2.5 hours in an ice-water bath. Imaging was performed with ChemiDoc MP imaging system (BIO-RAD) using Cy3 and Cy5 filters.

## 5. Exosome extraction

Exosomes were collected from the supernatant media of HT-29 cells (ATCC). Cells were first harvested with 5% FBS and Glutamax (Gibco) supplemented DMEM/F12 media in a 10 cm dish until they reached 80% confluency. The cells were passaged several

times, and expanded to 30 sets of dishes. Once confluency reached 100%, the media was discarded and cells were washed with PBS to remove BSA-derived EVs. DMEM/F12 media without FBS was then added to the dishes and cells were further cultured for additional 3 days. The media was collected and cellular debris was removed by filtration with a 0.2 μm filter. The filtered media was concentrated 100× using an ultrafilter (Amicon Ultra-15, NMWL:100K, Millipore) before being used for further experiments.

To obtain consistent reactivity for exosome capture using anti-CD9 antibodies, prepared exosomes were analyzed with Chemiluminescence Enzyme immunoassay (CLEIA) method. First, magnetic beads conjugated with anti-CD9 antibody (ExoCap™ CD9 Kit for Serum Plasm, MBL) were diluted to 0.1 wt% with a dilution buffer included in the kit. For 100 μL of this magnetic bead suspension, 1X exosomes diluted was added. Then, this mixture was incubated with six sets of incubation times (20, 60, 120, 180, 240, and 300 min). Each incubation was performed in triplicate. After incubation, Alkaline phosphatase-labeled anti-CD81 antibody (MBL, MEX003-3) was added to each solution and mixture was incubated for 20min. A chromogenic substrate (Lumipulse. FUJIREBIO) was added and the intensity of the luminescence was detected using a luminometer (Promega Glomax).

### 6. EV capture using DNA Tripods

For EV capture, 20 μL of DNA tripods (having anti-CD9 antibodies, DNA Origami concentration is around 2 nM) and 5 μL of 100 X exosome solution were mixed and incubated for 24 hours at room temperature. After this capture process, the mixture was subjected to agarose gel electrophoresis and TEM analysis. To evaluate the effect of ionic concentration on exosome capture, NaCl (final concentrations: 0, 25, or 100 mM) was

supplemented into incubation buffer.

## 7. TEM observation

Both DNA tripod-exosome complexes and exosomes are absorbed for 2 min on copper grids that were hydrophilized using glow-discharging. After deposition, the grids were stained with 2% aqueous Uranyl acetate solution containing 25 mM NaOH. Imaging was performed using H-7600 (Hitachi) and operated at 80–200 kV.

## 8. Single-particle imaging

Single-particle images were visualized by a total internal reflection fluorescence (TIRF) microscope equipped on an inverted type microscope (IX71, Olympus) with a ×60 oil immersion objective lens (UAPON 60×OTIRFM, NA 1.49, Olympus), as previously described[36-38]. Tripods and exosomes were labeled by Alexa647 (A647) dyes [using A647-streptavidin (SA) instead of A647-antibody (Ab)] and by SYTO RNASelect Green (Thermo), respectively. To prepare the observation chamber, we first infused SA into PEG/PEG-biotin coated quartz glass chambers. After washing with D-PBS (−), biotinylated capture strands for Tripod (capture strands can hybridized with anchor handle on the bottom of Tripod. In addition, capture strands bind to SA on the glass surface via biotin-SA binding) were infused into the chamber, and washed with D-PBS (−). Then, mixture of tripods and exosomes was infused into the glass chamber. After washing again with D-PBS (−), green (exosomes) and red (A647, tripods) dyes were illuminated with a DPSS laser (515nm; Fandango150, Cobolt) and a helium–neon (He–Ne) laser (633 nm; GLG5410, SOC), respectively. Green and red fluorescence images were separated by using a DualView2 (Optical Insights) and then projected side-by-side onto a back-

illuminated electron-multiplying charge-coupled device (EMCCD) camera (iXon3 DU-897E-CSO-#BV, 512 × 512 pixels, Andor Technology). Image analysis was performed using ImageJ with built-in functions and macros used in our previous paper[38]. Co-localization of tripods and exosomes was judged by eye.

## 9. Quantification of antibodies integrated onto DNA tripods (related to Supplementary Fig. 5)

The number of antibodies on DNA tripods was estimated using gel analysis. First, we estimated the label ratio of Alexa647 (A647) on an anti-CD9 antibody. The protein mole number of A647-labeled anti-CD9 antibodies in band 1 was estimated to be 0.55 pmole from actual loading amount in the lane. Assuming the number of A647 molecules on an anti-CD9 antibody was "X," the total amount of A647 in band 1 was estimated to be 0.55*X pmole. Whereas, protein mole numbers of A647-labeled streptavidin (A647-SA), which holds three A647 molecules based on product data sheet, in band 2 was estimated to be 1.1 pmole from actual loading amount in the lane. Thus, the total amount of A647 in band 2 was estimated as 1.1*3 = 3.3 pmoles. Moreover, the quantitative ratio of A647 band intensity for bands 1 and 2 was approximately 1:4.5 ~ 1:5. Thus, 0.55*X : 3.3 = 1 : (4.5 ~ 5), and the number of A647 molecules present on an anti-CD9 antibody was determined to be, X = 1.33 ~ 1.2.

Next, we determined the antibody number integrated on DNA tripods. The numbers of DNA tripods conjugated with A647-labeled antibodies mediated with non-labeled streptavidin in band 3 was found to be 41 fmole. Assuming the number of A647 present on a given DNA Tripod is "Y," total amount of A647 in band 3 was estimated to be 41*Y*(1.2 ~ 1.33). Whereas, the amount of A647 labeled SA in band 4 was 495 fmole.

Thus, the total amount of A647 in band 4 was estimated to be 495*3=1485 fmole. And the quantitative ratio of A647 in lanes 3 and 4 as determined using fluorescence was approximately 1: 9.4. Consequently, 41*Y*(1.2 ~ 1.33) : 1485 = 1 : 9.4, and Y was therefore estimated to be 2.9 ~ 3.2.

**10. Model particle capture (related to Supplementary Fig. 9)**

Streptavidin (SA) MicroBeads (50 nm: 130-048-102, Miltenyi Biotec) and 150 nm SA-magnetic beads (JSR) were mixed with 60° and 100° Tripods as follows. For 50 nm beads (concentration not provided by the supplier), 35 μL of beads were mixed with 20 μL of 2 nM Tripods. After 30 min incubation, samples were placed on a magnetic stand. After 1 day, the supernatant was discarded and fresh buffer (50 μL, 1x Origami buffer: 9 mM MgCl2, 5 mM Tris-HCl, 1 mM EDTA, pH = 8.0) was added and the samples were dispersed. After another day in a magnetic stand, the supernatant was discarded and fresh buffer (25 μL) was added and samples were imaged by ChemiDoc MP imaging system (BIO-RAD) using Cy3 and Cy5 filters. For 150 nm beads, 1 μL of 1% bead solution was dispersed in 50 μL of fresh buffer. After collection by magnetic stand, the beads were washed with 50 μL buffer and collected again. After dispersion with 30 μL fresh buffer, 2 nM of 20 μL Tripod was mixed and incubated for 2 days (to ensure an equivalent reaction time to that of treatment of 50 nm beads, as 50 nm beads need ~1 day for separation and overall required 2 days including wash step). After collection on a magnetic stand, the sample was washed twice with 50 μL fresh buffer, before dilution in 20 μL fresh buffer. Samples were imaged by ChemiDoc MP imaging system (BIO-RAD).

11. **Simulation analysis of the interaction between exosomes and DNA tripods**

**(related to Supplementary Fig. 10)**

In order to evaluate the size selectivity of multi-binder system, we performed a simple Monte Carlo simulation, where the stability of the Tripod-EV complexes formed was calculated based on a simple model (measuring the dissociation speed of EVs from Tripods). In this simulation, we assumed very simple situation, i.e., each binding site functions independently and there is no hysteresis for the release- and rebinding action of each binding site. Moreover, we assumed that antibody(es) does not dissociate from Tripod and that the number of antigen(s) on EV does not change during the simulation time. For the simulation, the outline of the algorithm is as follows:

1. Simulation starts with a single EV attached to the Ab-Tripods that have single-, double-, or triple binding sites (1-site, 2-site, and 3-site Tripods).

2. For each time step, repeat the following procedures for each binding site in random order.

i) If the binding site is currently attached to the antigen on EV, test the detachment and determine whether it remains in the attachment state or changes to the detachment state. The probability of detachment is defined by the parameter $k_{\mathrm{off}}$.

ii) If the motor is currently detached, test the attachment and determine whether it remains in the detachment state or changes to the attachment state. The probability of attachment to an antigen on EV is defined by the parameter: $k_{\mathrm{on}}$. In order to simplify the simulation, we assumed that the effective concentration of antigen on exosomes is 1 M [the average distance between antibody and target antigen on exosomes is roughly 1nm, using Avogadro's

number ($6 \times 10^{23}$) molecules in 1 L (= $(0.1\ m)^3$)].

3. If the termination condition is satisfied (all binding site(s) are in the detachment state), record the current time as the release time; otherwise, go to step 2.

We found that the key to high selectivity is 1) faster $k_{off}$ and 2) higher rebinding speed of each binding site. Faster $k_{off}$ contributes to accelerating the release of single- and double-binding site occupied EVs (i.e., smaller EVs than the designed aperture release faster compared to target-sized EVs that can simultaneously occupy three-binding sites, see the left panel of **Supplementary Fig. 10 b**). A higher rebinding speed contributes to an increase in the probability of EV retention (see left panel of **Supplementary Fig. 10 c**). A higher rebinding speed can be achieved by 1) the higher $k_{on}$ value of each binding site to the antigen on EVs and/or 2) the higher effective concentration of antigen on EVs. Since the geometry of binding sites and antigen on target EVs are beneficial for EVs with best fitted to the designed aperture (short distance ensures the effective collision and consecutive binding event between binding sites and antigen), the effective concentration of antigen on EV is higher for EVs with target size but not for smaller EVs, which contributes the size selectivity of our multi-binder approach.

With a higher $k_{on}$ value, the faster $k_{off}$ condition improves the selectivity at the expense of a decrease in the Tripod-EV complex (right panel of **Supplementary Fig. 10 c and d**). The balance between the selectivity and total Tripod-EV complex number will be decided based on the aim of the experiments and based on the situation of the sample (see next paragraph).

Other factors to consider in future studies:

1) Rebinding of the released EVs

In real experiments, the released EVs can rebind to Tripod and will affect the final distribution of bound EVs (see also below factor #5).

2) Force dependency of $k_{off}$

For smaller EVs, tension would be applied to each binding site-antigen interaction (since a pulling force is applied to each interaction), accelerating the release of smaller EVs.

3) Antigen density and its mobility on EVs

The density and mobility of the antigen will affect the effective $k_{on}$ to the binding site. Antigen selection will be important to increase the specificity especially to distinguish EVs from different sources.

4) Size distribution of EVs

Since the DNA origami scaffold can exclude larger particles, the fraction of smaller particles is more important in the DNA origami-based multi-binder system.

5) Ratio of Tripods and EVs

In addition to kinetic selection, competitive selection can improve the size selectivity of Tripod.

All in all, a faster $k_{off}$ and higher rebinding speed of each binding site ($k_{on}$ ) ensure the selectivity. Recent de novo protein design technology (including the 'generative' approach, ref 30 of the main text) will accelerate the development of binder having specific specs of $k_{on}$ and $k_{off}$. The small size (50 amino acids (a. a.) ~. C.f. nanobody is around 150 a. a.) of the de novo designed protein will be beneficial to integrate multiple binders with high density due to the reduced space occupation. Combined with the

versatile scaffold design capability of DNA origami, the multi-binder system will contribute significantly to biology and other fields.

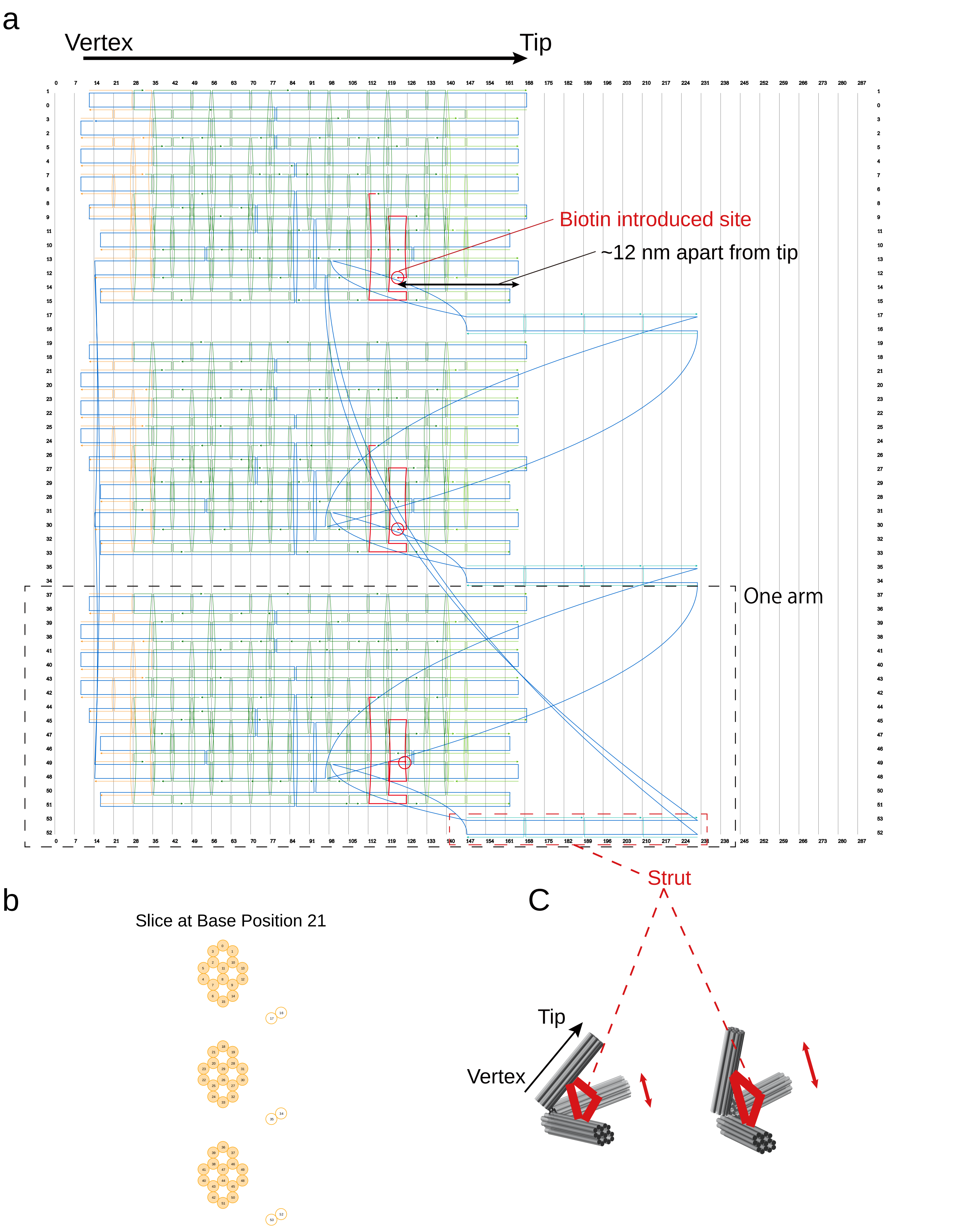


**Supplementary Fig. 1. Design of DNA tripod.**
(a) Secondary structure of the DNA tripod rendered using caDNAno. Red open circle indicates the position of biotin. Strut region that defines the angle of Tripod is highlited with red dashed line. (b) Cross section image of caDNAno. (c) Strut length change makes the apature (angle) change of Tripod.

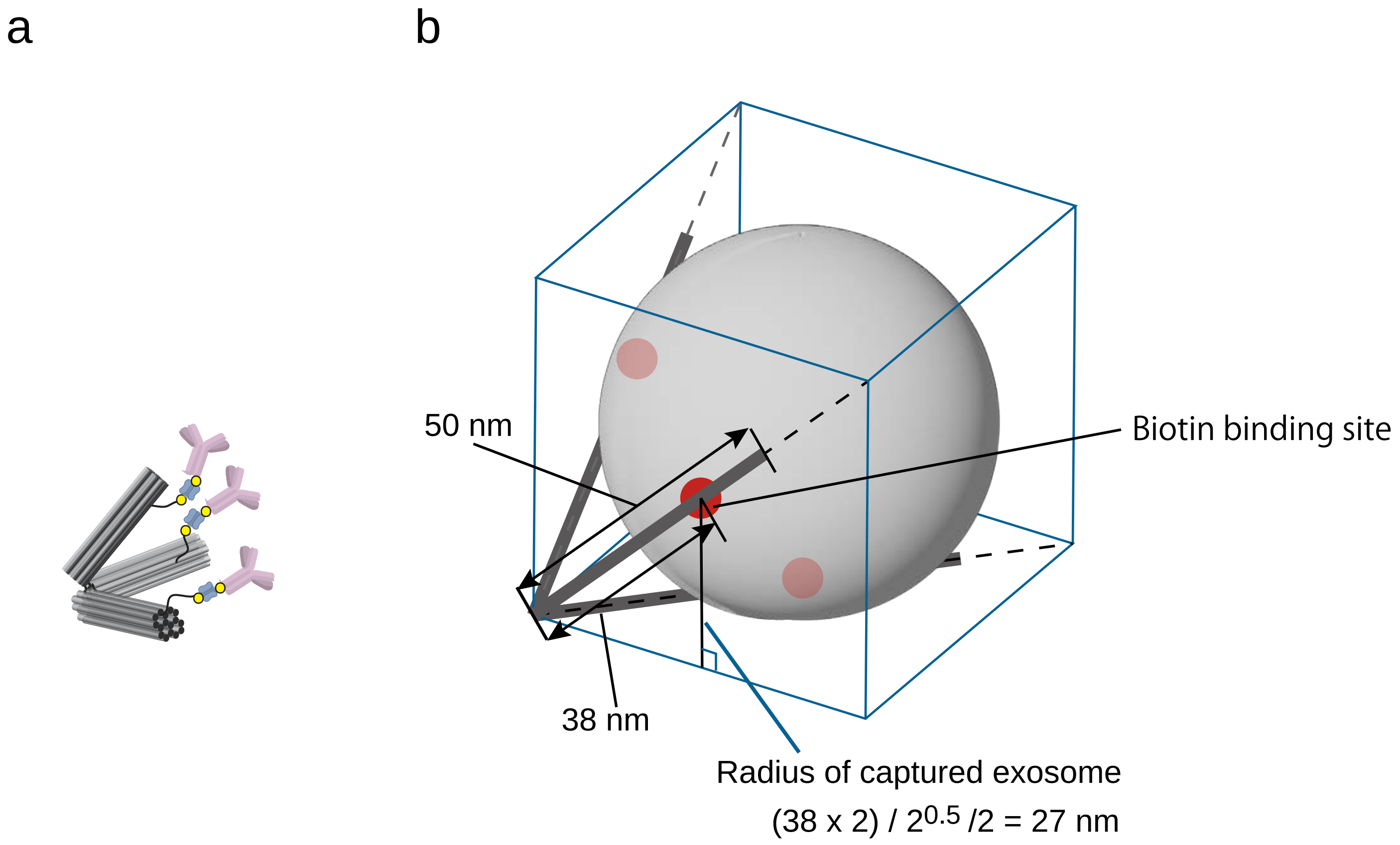


**Supplementary Fig. 2. Predict the captured exosome size based on Tripod geometry.**
(a) Schematic illustration of the Tripod structure. (b) In order to evaluate the exosome size that can be captured by Tripod, we assumed a simple case, i.e., Tripod arms are rigid and the exosomes are perfect spheres and also rigid. In this case, the geometric calculation provides the minimum (defined by the binding site geometry) and maximum (defined by the arm tip geometry) values of the captured exosomes. We first consider the 60° Tripod. The sphere that can be bound inside the 60° Tripods is the same size as the sphere that is in contact with a regular tetrahedron. The binding site of Tripod to the exosome (e.g., biotin integration site) is the point of tangency. Therefore, based on the geometry, we can calculate the radius of the sphere to be $a / 2^{0.5}$, where $a$ is the arm's length from the vertex to the point of tangency, i.e., the biotin integration site (binding site). If we take 38 nm for $a$, the radius of the sphere can be calculated as 27 nm and the diameter is 54 (= 27 × 2) nm. Similarly, we can calculate the maximum sphere size that can maintain inside Tripod. In this case, we take 50 nm (= maximum arm length) as $a$ and obtain 71 (= 50 / 2^0.5 × 2) nm. Thus, the 60° Tripod can capture spheres of 54–71 nm, if the antibody can access the surface of the exosome.

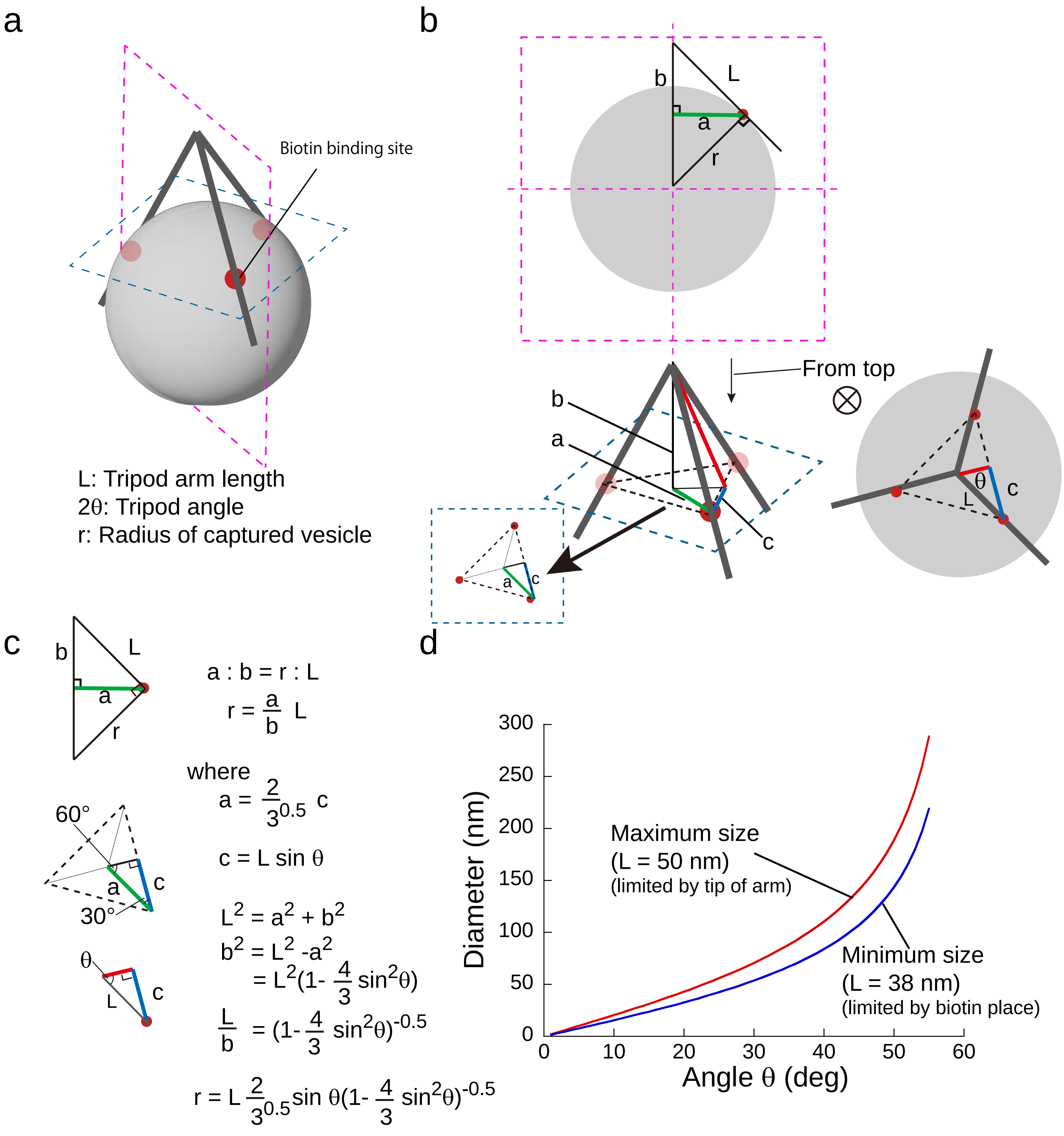


**Supplementary Fig. 3. Predict the captured exosome size based on Tripod geometry (2).** (a) Schematic illustration of Tripod structure. Dashed Region Of Interests (ROIs) indicate cross-section. Red circle indicates biotin binding site. (b) To generalize the prediction of captured size, we set parameters: *L*: Tripod arm length; 2θ: Tripod angle; **r**: Radius of captured vesicle. To calculate *r* we set parameter *a*, *b*, *c*. (c) From the geometory *r* can be calculated. (d) Tripod angle dependency of diameter of captured vesicle. Maximum size of captured vesicle is limited by the tip of the Tripod arm (L = 50 nm). Minimum size of captured vesicle is limited by arm length from vertex to biotin binding site (L = 38 nm in this study).

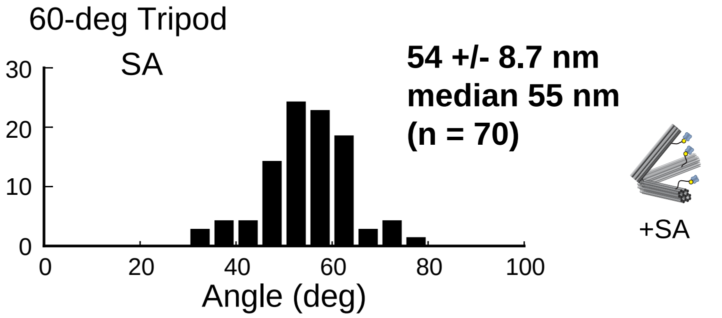


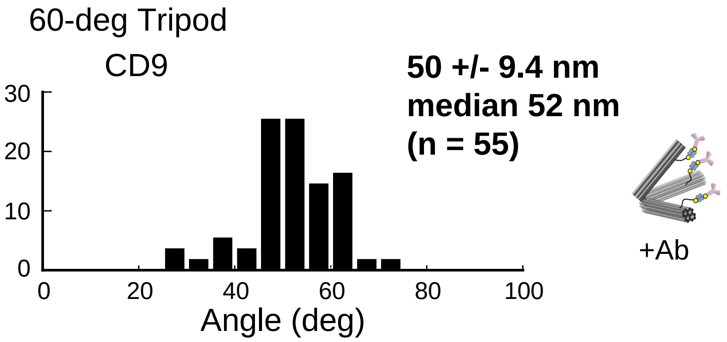


**Supplementary Fig. 4. Angle distribution of 60° Tripod.**
Strept-avidin (SA) conjugated Tripods, SA-Tripods (uppder), antibody-conjugated Tripods, Ab-Tripods (lower), were imaged by electron microscope (negative stain). And angle between arms were measured.

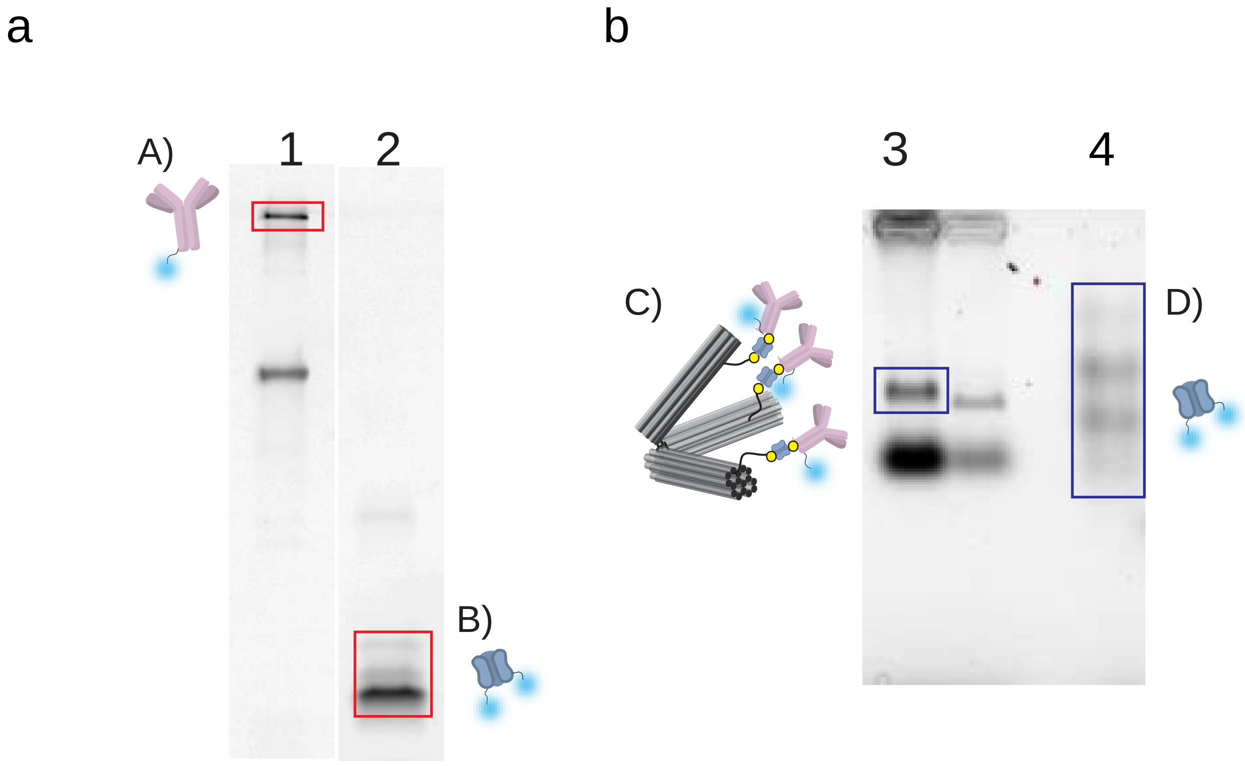


**Supplementary Fig. 5. Quantification of antibodies integrated on DNA tripods.** (a) Estimation of Alexa647 (A647) label ratio of anti-CD9 antibody using SDS-PAGE. Fluorescent intensity of region of interest (ROI) A in lane 1 was compared with that of ROI B (lane 2) of A647-streptavidin (SA). (b) Estimation of the number of anti-CD9 antibodies integrated on DNA tripods using agarose gel. Fluorescent intensity of A647-labeled anti-CD9 antibodies (ROI C in lane 3) was compared to that of A647-SA (ROI D in lane 4). Please see Supplementary Methods for detail.

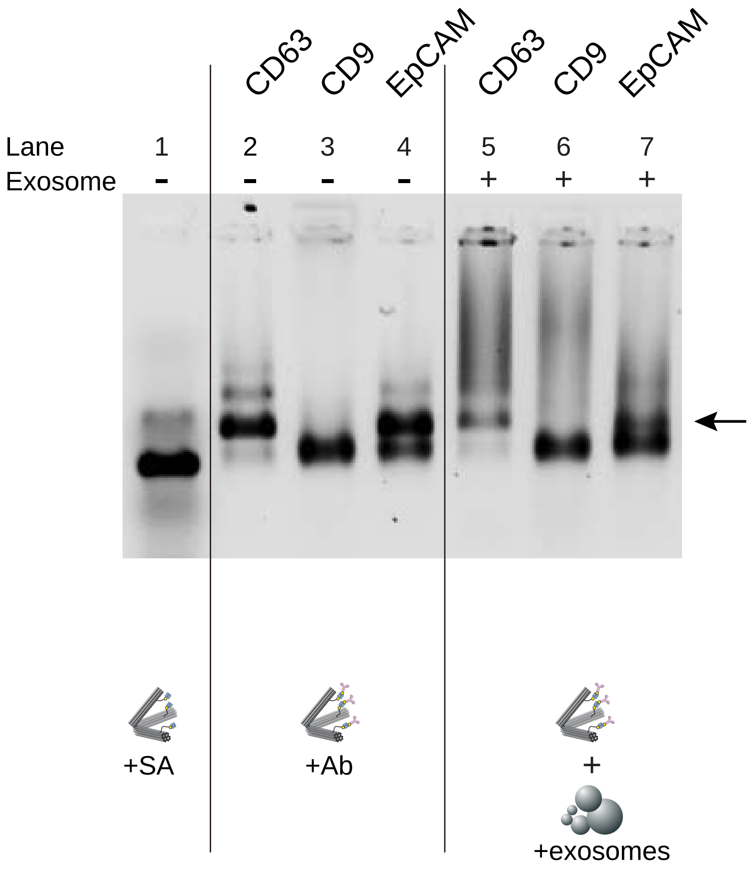


**Supplementary Fig. 6. Optimization of the antibody.**
Strept-avidin (SA) conjugated Tripods, SA-Tripods, were mixed with antibodies (anti CD63, CD9, EpCAM), and after purification of antibody-conjugated Tripods, Ab-Tripods, exosome (indicated by "+" in the figure) were incubated with Ab-Tripods. The smear band that appeared in the exosome-adding condition suggests the binding of Ab-Tripods to exosomes. The binding efficiency , in our hand, was CD63 > CD9 > EpCAM. Moreover, CD63- and EpCAM Ab-Tripods showed dimer Ab-Tripods (indicated by arrow). Taken together, we chose CD9 for this study. In the case of other target exosomes with different origins (birthplaces) other than this study, the optimal antibody would change.

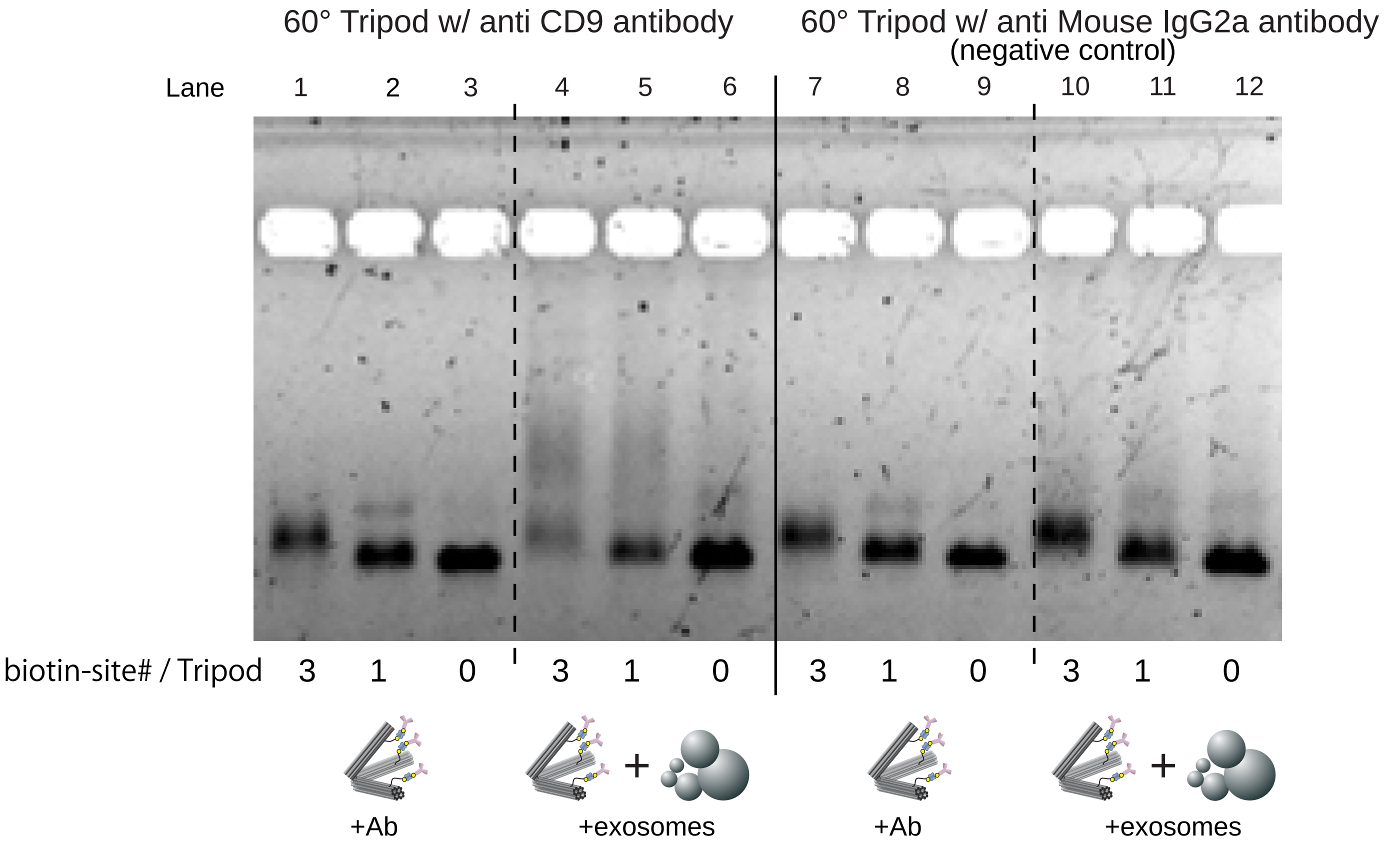


**Supplementary Fig. 7. Effect of biotin site number per Tripod.**
The binding amount of 60°-60°-60°DNA tripods was estimated using agarose gel electrophoresis. Lanes 1–3: DNA Tripod / Streptavidin / Anti-CD9 antibody (biotin handle 3, 1, 0); Lanes 4–6: + exosome (biotin handle 3, 1, 0. Bound fractions were 59 and 26% for 3 and 1-antibody Tripod); Lanes 7–9: DNA Tripod / Streptavidin / Anti-Mouse IgG2a antibody (biotin handle 3, 1, 0); Lanes 10–12: + exosome (biotin handle 3, 1, 0).

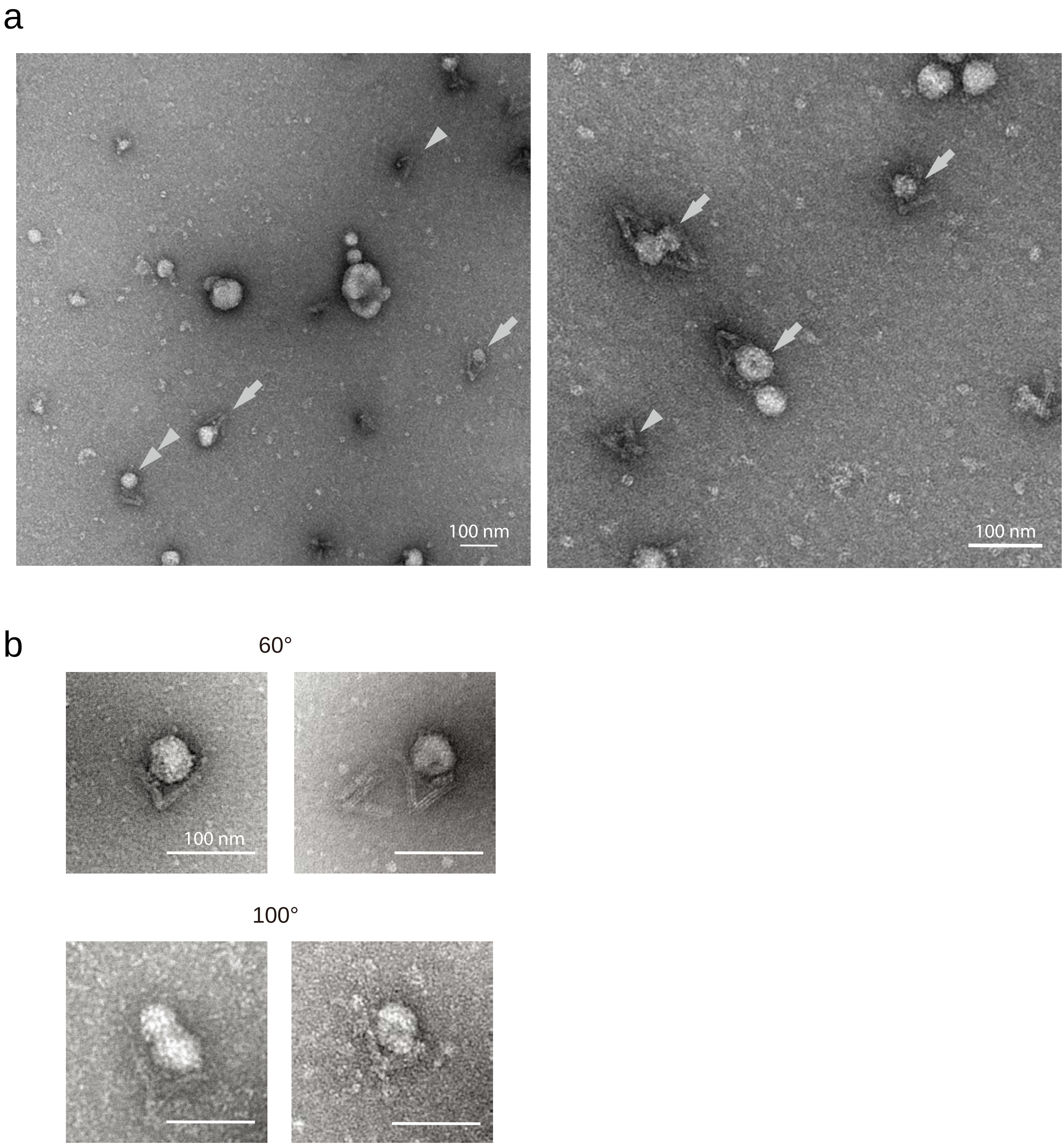


**Supplementary Fig. 8. Additional TEM images of reaction mixture of EVs and Tripods.**
(a) Wide-view images of reaction mixture of EVs and 60°-60°-60° Tripods. Arrows indicate exosomes captured by tripods, arrowheads indicate empty tripods, and double arrowheads indicate exosomes near the tripods. (b) Additional typical TEM images of EV-Tripod complex for 60° Tripods (upper) and for 100° Tripods (lower).

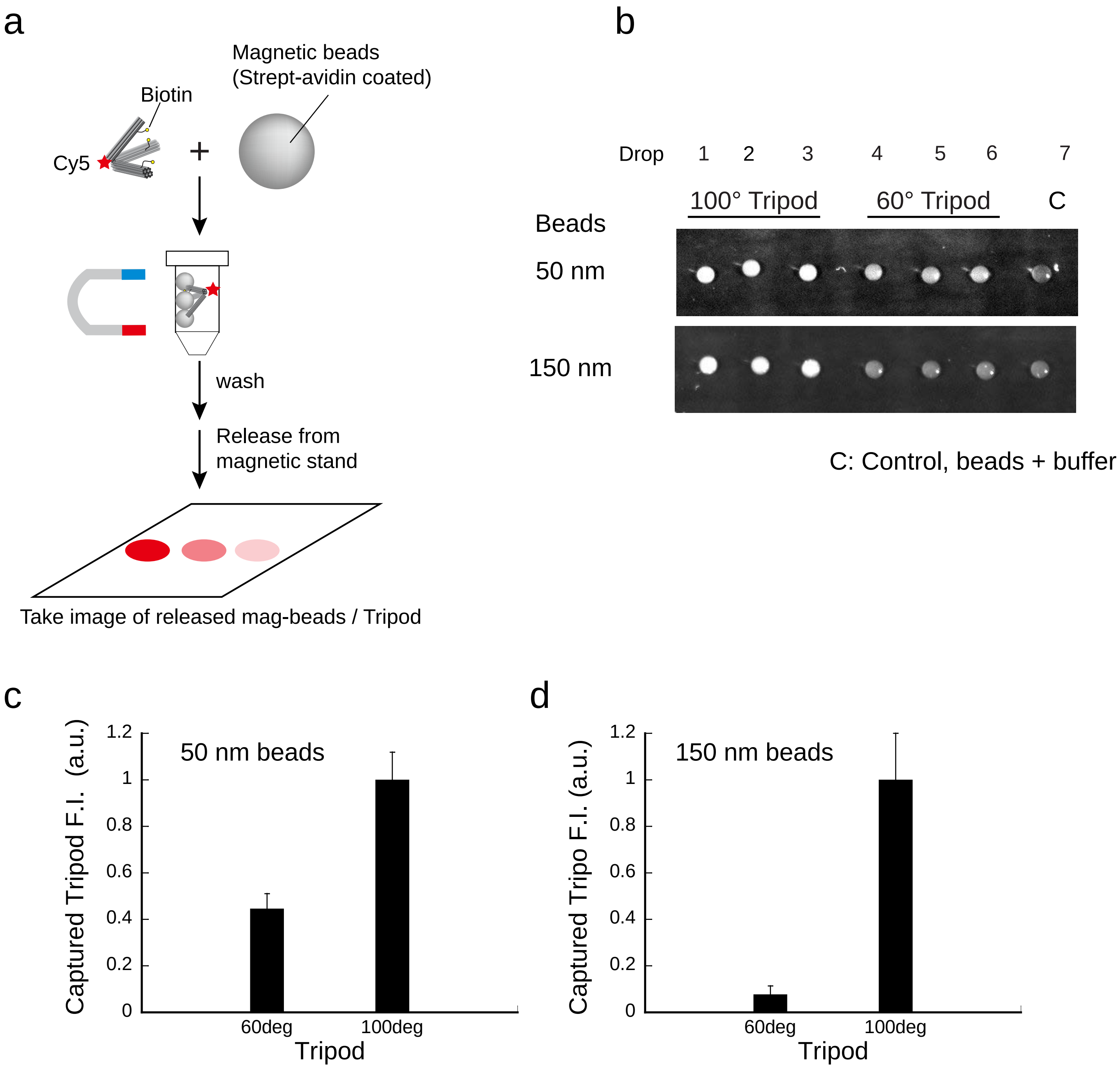


**Supplementary Fig. 9. Capture of the model particle.**
(a) Schematic illustration of the experiments. Tripods with biotin/Cy5 were prepared and mixed with strept-avidin (SA)-coated magnetic beads (diameter of 50 and 150 nm). After incubation, the magnetic beads were captured by a magnetic stand. After the washing process, the remaining magnetic beads were released from the magnetic stand and imaged. (b) Drop image of released magnetic beads. If Tripods can capture magnetic beads, the Cy5 signal of Tripods can be observed. As expected, 50 nm beads can be captured both by 60° and 100° Tripods, but 150 nm beads can only be accessed by 100° Tripods, but not by 60°. Each drop is an independent experimental sample. (c,d) Quantification of (b). For each graph, data of 100° for 50 and 150nm beads were set to 1 for (c) and (d), respectively.

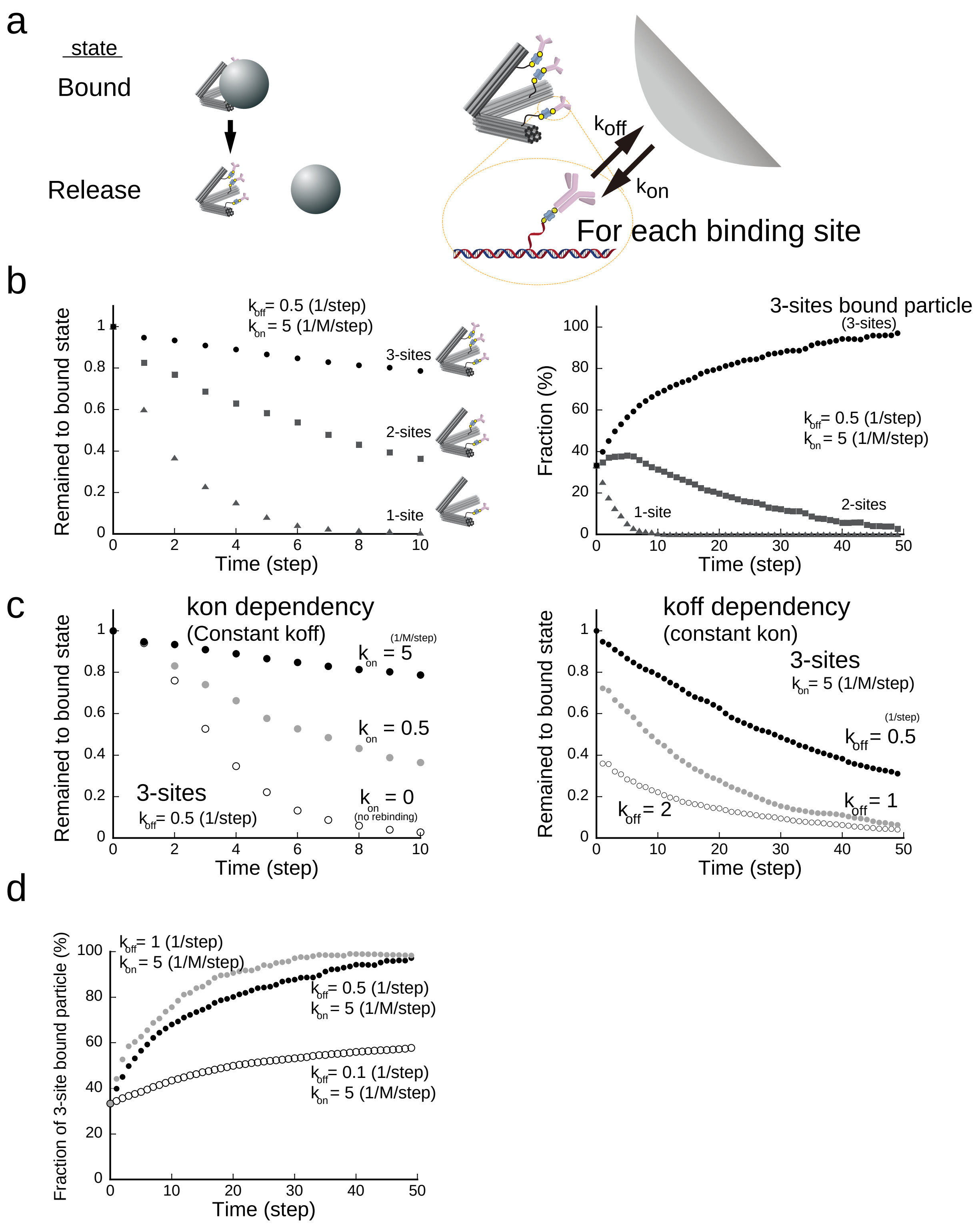


**Supplementary Fig. 10. Simulation analysis of the interactions between exosomes and DNA tripods.** (a) (left) Schematic illustration of the simulation. The stability of Tripod–EV complexes was analyzed using the Monte Carlo method. (right) Attachment and detachment events of each binding site of Tripod were calculated (see Supplementary Materials and Methods for details). (b) (left) Effect of binding site number. Tripods with 1, 2, or 3 sites were compared with fixed kinetic parameters [koff = 0.5 (1/step), kon = 5 (1/M/step)]. (right) The fraction of 3 site bound particles increases with time, indicating that the smaller particles dropped (release) with time, and the selectivity reaches nearly 100% with time. (c) Dependency of kon (left) and koff (right). Higher kon will increase the fraction remaining through the occasional rebinding events (left). Faster koff decreases the total number of Tripod-EV complexes with time. (d) Faster koff contributes to faster selectivity. In order to simplify the simulation, we assumed that the effective concentration of antigen on exosomes is 1 M (the average distance of the molecule is 1 nm. See Supplementary Materials and Methods for details).

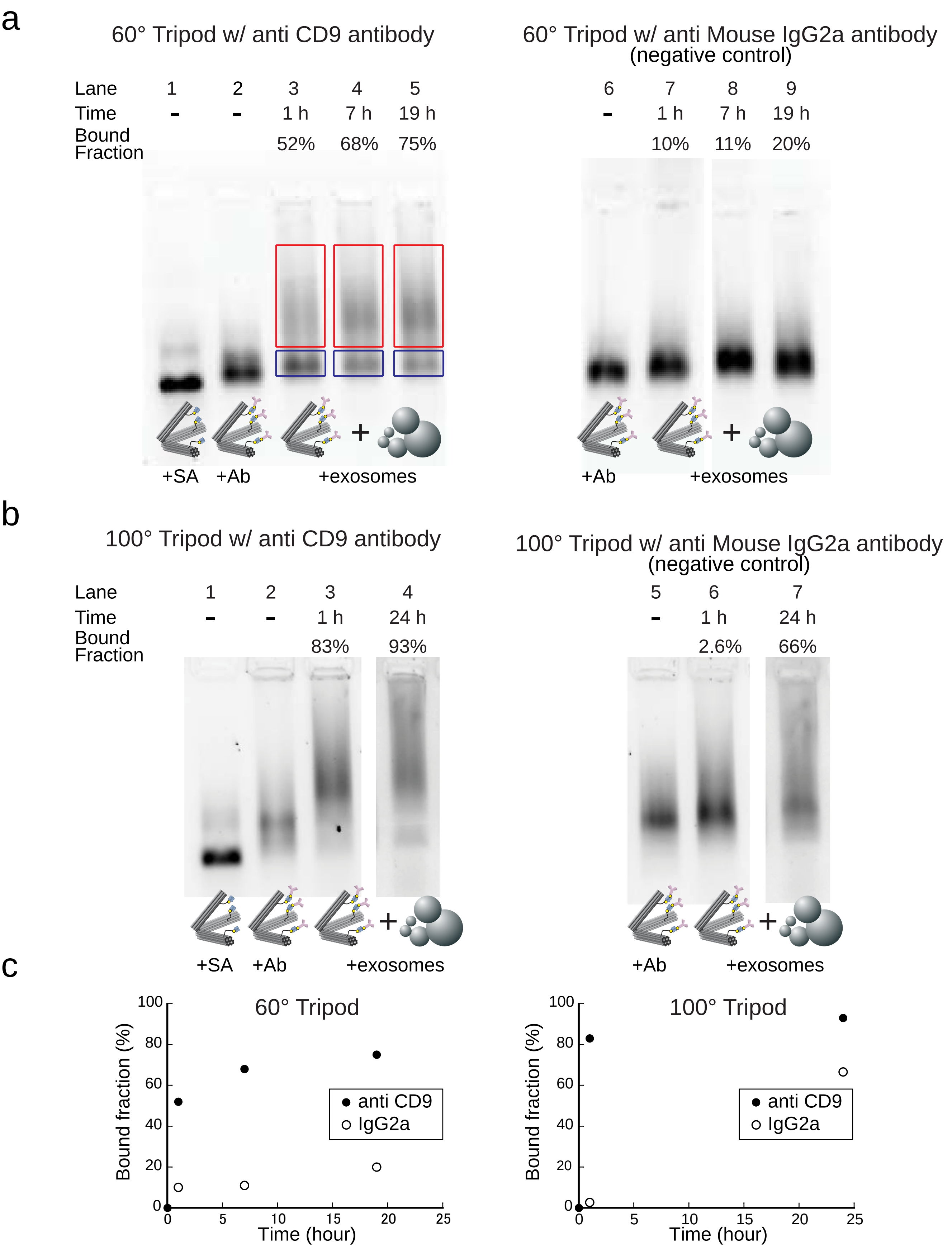


**Supplementary Fig. 11. Kinetic analysis of interactions between exosomes and DNA tripods.** (a,b) Binding speed of 60°–60°–60° (a) and 100°–100°–100° (b) DNA tripods were estimated using agarose gel electrophoresis. Fluorescent intensities of exosome-bound (red ROI) and unbound DNA tripods (blue ROI) were used to estimate the bound fraction of DNA tripods. (a) Lane 1: DNA Tripod / Streptavidin, Lane 2: DNA Tripod / Streptavidin / Anti-CD9 antibody, Lane 3-5: + exosome (1, 7, 19h incubation), Lane 6: DNA Tripod / Streptavidin / Anti-Mouse IgG2a antibody, Lane 7-9: + exosome (1, 7, 19h incubation). (b) Lane 1: DNA Tripod / Streptavidin, Lane 2: DNA Tripod / Streptavidin / Anti-CD9 antibody, Lane 3&4: + exosome (1, 24h incubation), Lane 5: DNA Tripod / Streptavidin / Anti-Mouse IgG2a antibody, Lane 6&7: + exosome (1, 24h incubation). (c) Quantification of (a) and (b).

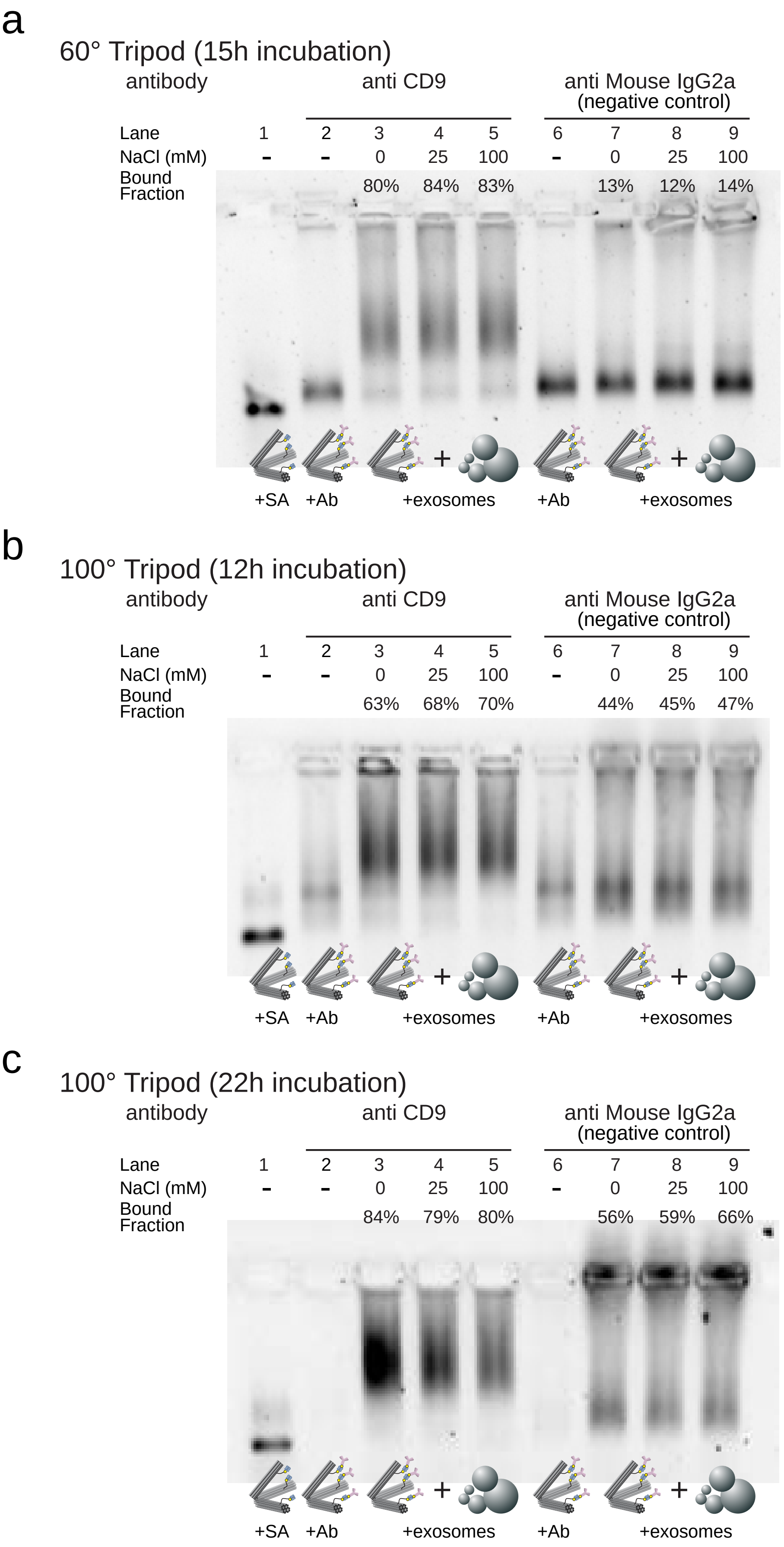


**Supplementary Fig. 12. Effect of salt (NaCl) on the capture process.**
(a-c) Effects of salt (NaCl) on the capture process of 60°–60°–60° DNA Tripods (a, 15 h incubation) and 100°–100°–100° (b and c) DNA tripods were estimated using agarose gel electrophoresis.
We note that bands of lanes 2 and 6 of (c), condition of DNA Tripod / Streptavidin / Anti-IgG2a antibody are weak, presumably because of loading error.

**Supplementary Table 1 | DNA oligonucleotide sequences of the handles used for anchoring and labeling.**

See also ref 14 (Iinuma R. *et al*. *Science*. 344, 65–69 (2014)) for detail staples.

B, biotin; dB, dual biotin; hybridization site is underlined for anchoring Tripod on glass surface

| Tripod | Fixing Target | Sequence | Position Of moiety |
|---|---|---|---|
| 60° | streptavidin-antibody | TTTCCTTTGCCCGAACGATCATATTATACTT AAATttttttt-B | 12[122] |
| 60° | streptavidin-antibody | TAAGTAACAACCCGTCGCCGTGCACAGCC AGGAGAttttttt-B | 30[122] |
| 60° | streptavidin-antibody | TAAGGCTGAGACTCCTCTATAGCCCCGCCA CTCAGCTTGGCTTAGttttttt-B | 49[125] |
| 100° | streptavidin-antibody | CCACCTCAATCCGTCAATAGATAACAAACA AGGAGCGGGAAAttttt-B | 12[143] |
| 100° | streptavidin-antibody | AAAAAATTCGACATTAAATGTGAGCAAAC GGTCAGGAACCCGttttt-B | 30[143] |
| 100° | streptavidin-antibody | ATATGCCTATTTCGGAAGCTGAGAGGTGTA TACTAttttt-B | 48[143] |
| 60° | Hybridize to capture strand on glass surface | TTTTTATTCCACACAACGCATTAATGAATC GGCCAATTTTT<u>TAACATTCCTAACTTCTCATA</u> | 6[9] |

| | | | |
|---|---|---|---|
| 60° | Hybridize to capture strand on glass surface | CGAAGCTGGCTAGTGAATGTAGTAAAACGAACTAACGGAACAACTTTTTTAACATTCCTAACTTCTCATA | 32[16] |
| 60° | Hybridize to capture strand on glass surface | TTTTTATTATTACAGGTGACGACGATAAAAACCAAATTTTTTAACATTCCTAACTTCTCATA | 24[9] |
| 60° | Hybridize to capture strand on glass surface | TTTTTCCGGTATTCTAAACGAGCGTCTTTCCAGAGCTTTTTTAACATTCCTAACTTCTCATA | 42[9] |
| 60° | Hybridize to capture strand on glass surface | TTTTTCACCCTGAACAATTAAGAAAAGTATTTTTTAACATTCCTAACTTCTCATA | 38[9] |
| 60° | Hybridize to capture strand on glass surface | TTTTTTTGATGGTGGTTCGAAAAACCGTCTTTTTTAACATTCCTAACTTCTCATA | 2[9] |
| - (glass Surface) | Hybridize to Handles on Tripod | dB-TATGAGAAGTTAGGAATGTTA | - |
| 60° | Cy5 labeling | TTTTTATTATTACAGGTGACGACGATAAAAACCAAAttttt-Cy5 | 24[9] |
| 60° | Cy5 labeling | TTTTTTTGATGGTGGTTCGAAAAACCGTCttttt-Cy5 | 2[9] |
| 100° | Cy5 labeling | TTTTTTCCGCTCACAATCGTGCCAGCTGCATTAATGttttt-Cy5 | 6[9] |
| 100° | Cy5 labeling | TTTTTGAAAAATCTACGCATAGTAAGAGCAACACTAttttt-Cy5 | 24[9] |
